# Attitudes towards chiropractic: a survey of Canadian sport and exercise medicine physicians

**DOI:** 10.1186/s12998-025-00581-4

**Published:** 2025-05-20

**Authors:** Cameron Borody, Janet D’Arcy, Jaime Waters, Mark Leung, Jason Busse

**Affiliations:** 1 Canadian Memorial Chiropractic College, 6100 Leslie St, Toronto, ON M2H 3J1 Canada; 2 The Open University, Milton Keynes, United Kingdom; 3 University of Toronto, Toronto, Canada; 4 McMaster University, Hamilton, Canada

**Keywords:** Chiropractic, Attitude, Sports medicine, Survey

## Abstract

**Background:**

Previous surveys of physicians revealed diverse attitudes towards chiropractic. One of several strategies proposed by leaders in chiropractic to support the advancement of the profession in North America is to increase opportunities for interprofessional collaboration. One area where chiropractic has achieved some degree of integration in Canada is in the field of sports medicine. A result of the increased integration of chiropractic in the field of sports medicine has been more opportunity for interprofessional collaboration, development of interprofessional relationships and increased exposure to chiropractic. The attitudes of Canadian sport and exercise medicine physicians (CSPs) towards chiropractic are unknown. The purpose of this study is to determine the attitudes and their contributing factors of CSPs toward chiropractic and its use for treatment of athletes and/or Canadians who are participating in sports or exercise (ACSE).

**Methods:**

An invitation to complete the survey was included in a newsletter emailed to active physician members of CASEM (Canadian Academy of Sports and Exercise Medicine) in March and April 2023 and attendees of their symposium. The survey included the Chiropractic Attitude Questionnaire (CAQ), which allowed respondents to indicate their attitudes towards chiropractic care for ACSE using a 5-point Likert scale. The responses to the CAQ were the primary outcome measure. Descriptive statistics, including mean, median, standard deviation, maximum, minimum and range, regression analysis, t-tests, and ANOVAs were used to analyse the data.

**Results:**

Seventy CSPs completed the survey (response rate: 11%). The summed CAQ scores ranged from 0 to 68 with a mean of 39.03 and a standard deviation of 15.30. CSPs who worked with a chiropractor in a multidisciplinary setting hold a more positive opinion of chiropractic. An independent samples t-test indicated that there was a significant difference between CAQ scores for those with experience of working with a chiropractor (M = 42.03, SD = 14.99) and those without (M = 29.41, SD = 13.10); t(58) = 3.27, *p* < 0.05.

**Conclusions:**

CSPs attitudes toward chiropractic and its use for treatment of ACSE range from very positive to extremely negative. CSPs who reported to have worked with a chiropractor have more positive attitudes than those that have not.

**Supplementary Information:**

The online version contains supplementary material available at 10.1186/s12998-025-00581-4.

## Background

In 2006, a strategic planning conference for the chiropractic profession made several recommendations including prioritizing the development and implementation of a strategy to increase interprofessional collaboration [1]. In Canada there is some evidence of progress in this regard with the inclusion of chiropractors in some Family Health Teams, working as Advanced Practice Practitioners screening patients for spine surgeons and the integration of chiropractic into some private sector medical facilities [2–6]. Sports medicine is one field where, in Canada, chiropractors have achieved some level of integration. In the pursuit of excellence in athletic performance, sports medicine has evolved to embrace an interdisciplinary model of care commonly referred to in Canada as an Integrated Support Team (IST) that often includes a chiropractor [7]. Interviews of stakeholders on sports medicine healthcare teams has suggested chiropractors’ inclusion was a manifestation of ‘consumer-focused practice’ and that ‘the athlete’s wanted them’ not necessarily that the profession had been accepted by other healthcare professionals [8]. Nonetheless, chiropractors have continued to integrate into the field of sports medicine. In 2010, at the Winter Olympics in Vancouver, chiropractic was included for the first time as an equal partner in the delivery of healthcare to all participants. It is now very common in Canada, particularly in urban centres, for private multidisciplinary clinics marketing themselves as “sports medicine” to include a chiropractor. The sports and exercise medicine residency programme at the University of Toronto has included a clinical placement with a chiropractor for its residents since 2014. At the 2023 Pan American Games in Chile, the Canadian Medical Services Team included 53 health care providers, of which five were chiropractors. In Canada, the sports medicine landscape has allowed for various opportunities for chiropractors to collaborate with Canadian sport and exercise medicine physicians (CSPs). Understanding how CSPs view chiropractic may provide information to examine the impact of improved integration, opportunities to enhance interprofessional collaboration and ultimately improve patient care. This study aims to evaluate the attitudes (hypothesis 1), and their contributing factors (hypotheses 2–4), of CSPs towards chiropractic.

## Methods

We adapted a questionnaire previously used to evaluate attitudes and beliefs of orthopedic surgeons towards chiropractic for our study [9]. Specific modifications include more details pertaining to medical training and practice experience resulting in a 49-item questionnaire that examined the attitudes of CSPs towards chiropractic care of athletes and/or Canadians who participate in sports or exercise (ACSE) (Appendix-A). The questionnaire included a 20-item section, the Chiropractic Attitude Questionnaire (CAQ), which captured respondent’s attitudes towards chiropractic. The remaining 29 items related to demographics and other professional characteristics and activities. The clinimetric properties of the CAQ were established in previous studies as part of its original development [9–11]. Respondents were invited to enter written comments in a final open-ended component at the end of the survey.

The survey was reviewed independently by ML and two residents in the enhanced skills sports and exercise medicine residency programme at the University of Toronto for suitability and relevance to their profession.

### Subjects

The inclusion criteria of this study consisted of medical doctors who are licensed and active members of the Canadian Academy of Sport and Exercise Medicine (CASEM) in any Canadian province or territory.

### Procedures

Respondents were CSPs that received the survey through distribution to the CASEM database and/or attended the 2023 CASEM symposium. We approached CASEM and they agreed to include an invitation to participate in our survey in their monthly newsletter. The invitation included a link to the online survey where participants were presented with a disclosure letter and the questionnaire. We used Survey Monkey to facilitate online completion of the questionnaire. The study disclosure letter notified potential participants of the purpose of the study, what their involvement would entail, and the risks and benefits of partaking in the study, as well as a disclaimer notifying them that they were not obligated to participate. If a potential participant agreed to the terms of the study they indicated as such by clicking on an “I agree” button that linked them to the survey. Only actively practicing CSPs were asked to complete the survey.

CASEM included the invitation to participate in our study in their electronic newsletter twice. It was sent to all 1045 CASEM members in March and April of 2023. At that time their membership included 638 active CSPs, which was our target population. CASEM provided us with some analytics from the Mail Chimp online distribution platform showing that in March and April, 289 and 280 recipients of the email clicked on the link to the newsletter that contained our invitation.

CASEM also agreed to include a card with a QR code linking to our survey in the lanyards for the 381 CSPs who attended the 2023 CASEM symposium in Banff, Alberta. The survey was available to potential respondents for three months.

There was no identifying information to link the respondent to a specific questionnaire and due to the nature of the software respondents were only able to complete the questionnaire once. Because Survey Monkey takes the IP addresses of respondents a research assistant stripped that information from the completed surveys prior to the research team reviewing the data. Respondents were asked if they were interested in the survey results and were prompted to provide their email address so that a summary of the study could be distributed to them upon completion of the study.

There was no compensation for participating in the study however respondents were asked if they would like to submit their email address to be included in a random draw for one of five $50 gift cards. A research assistant removed the email addresses from the surveys of those that submitted for the random draw and after the data collection period was completed a randomization software programme was used to identify five email addresses to award a gift card.

### Outcome measures

The primary outcome measure for this study was the CSP’s attitudes towards chiropractic and its use for treatment of ACSE as determined by the total score on the CAQ.

### Analysis

Each of the 20 questions comprising the CAQ was graded on a 5-point Likert scale, from 0 to 4. The responses were then summed to arrive at a total score ranging from 0 (most negative attitude towards chiropractic) to 80 (most positive attitude towards chiropractic). Demographic frequencies for gender identity, years in practice, and previous work experience were calculated. For the evaluation of the hypotheses listed below a statistical test was considered statistically significant if *p* < 0.05. Written comments provided by respondents will be evaluated in a separate analysis.

#### Hypothesis 1

CSPs attitudes towards chiropractic will be diverse. We conducted an inspection of the CAQ scores including mean, median, standard deviation, maximum, minimum and range. We also carried out a regression analysis to explore the factors influencing attitudes towards chiropractic. A generalized linear model was employed, with the aggregate CAQ score serving as the dependent variable. Selection of the influencing factors was based on previous studies in this area [9–11] and CSP demographics. All independent variables were nominal except for ‘number of patients referred for chiropractic care in a typical year,’ which was ordinal (ranked groups from ‘0’ to ‘50 + ’). Comparisons were all two-tailed, and variables were deemed statistically significant if the p-value was less than 0.05 in the final multivariable model. The unstandardized regression coefficient and its 95% confidence interval (CI) are reported, where the unstandardized regression coefficient indicates the change in the CAQ response score. Multicollinearity was deemed problematic if the variance inflation factor for any independent variable exceeded 5 [12] however this was not the case for any of the variables. All analyses were conducted using IBM SPSS Statistics software (version 29).

#### Hypothesis 2

CSPs who currently work with or have worked with a chiropractor in a private community-based clinic or as members of a specific IST with a National Sport Organization (NSO) or as part of the medical team at a multisport games will hold a more positive opinion of chiropractic. We tested this hypothesis in two ways. First, we combined all responses that confirmed previous work experience with a chiropractor in any of the specific scenarios detailed above and applied a t-test to investigate the relationship between previous experience working with a chiropractor (group/independent variable) and the CAQ score (test/dependent variable). Secondly, we applied an ANOVA test where each of the three different scenarios where respondents have previous experience working with a chiropractor or no previous experience working with a chiropractor to investigate the relationship between the different work experience scenarios (group/independent variable) and the CAQ score (test/dependent variable).

#### Hypothesis 3

CSPs indicating a favourable relationship with a specific chiropractor will hold a more positive opinion toward chiropractic. We applied a t-test to investigate the relationship between CSPs who indicated a favourable relationship with a specific chiropractor (grouping/independent variable) and the CAQ score (test/dependent variable).

#### Hypothesis 4

CSPs who indicate they had exposure to chiropractic during their sport and exercise medicine residency programme will hold a more positive opinion toward chiropractic. We applied an ANOVA test to investigate the relationship between the different exposure scenarios (Yes, overall favourable, yes, overall neutral, yes, overall unfavourable and no exposure) and the CAQ score.

## Results

The link to the survey was sent to all 638 active physician members of CASEM. Eighty-two recipients responded (13%) and 70 completed the survey (response rate: 11%). Although the entire population was included in the sampling frame, due to the low response rate and self-selecting nature of participation, overall generalizability of results is unclear.

### Demographics

Participant demographics were examined in terms of gender identity, years in practice, and previous work experience (see Table 1). Over half of CSPs (38 of 70, 54.29%) had worked with a chiropractor in a multidisciplinary setting, and over two-thirds (48 of 70, 68.57%) had referred at least one patient to a chiropractor in the last year.

**Table 1:** Demographic characteristics of respondents

No. of respondents	70
Gender, n (%)	
Male	34 (48.57%)
Female	35 (50.00%)
Other	1 (1.43%)
Years in practice, n (%)	
< 5 yr	23 (32.86%)
5–10 yr	12 (17.14%)
11–20 yr	8 (11.43%)
> 20 yr	27 (38.57%)
Practice environment, n (%)	
Community	44 (62.86%)
Hospital-based	17 (24.29%)
Multidisciplinary	17 (24.29%)
Private practice	30 (42.86%)
Academic	16 (22.86%)
Other	3 (4.29%)
Clinical area of interest, n (%)	
Family medicine	26 (37.14%)
Occupational medicine	5 (7.14%)
Emergency medicine	10 (14.29%)
Sports medicine	64 (91.43%)
Psychotherapy/Psychiatry	4 (5.71%)
Pediatrics	8 (11.43%)
Pain medicine	9 (12.86%)
Orthopaedics	15 (21.43%)
Physiatry	11 (15.71%)
Other	8 (11.43%)
Experience working with a chiropractor, n (%)	
Yes	38 (54.29%)
No	22 (31.43%)
No, but open to working with a DC	10 (14.29%)
Setting of work with a DC, n (%)	
Integrated Support Team (National Sport Organization)	16 (42.11%)
Professional Sports Team	11 (28.95%)
At a multisport games (i.e. Olympics, Pan Am Games)	20 (52.63%)
At a single sport competition (i.e. National Championship)	15 (39.47%)
Community-based sports medicine clinic	25 (65.79%)
Other	3 (7.89%)

#### Hypothesis 1

Just under half (45.71%) of CSPs had an overall positive impression of chiropractic, compared to 30.00% who had a negative view, and 24.29% whose overall impression was neutral. Individual responses for each of the twenty questions can be found in Table 2. Most questions did not elicit a particularly strong responses one way or the other, with the exception of ‘Chiropractors provide effective therapy for some musculoskeletal conditions’ where the majority (75.71%) of CSPs agreed/strongly agreed and ‘Chiropractors can provide effective therapy for some non-musculoskeletal conditions (e.g. asthma, infantile colic), where the majority (77.14%) of CSPs disagreed/strongly disagreed. Overall, the summed CAQ score ranged from 0 to 68 with a mean of 39.03 and a standard deviation of 15.32. Breaking this down, those with a positive overall impression of chiropractic had a mean CAQ score of 50.78 (SD = 7.36), while an undecided overall impression of chiropractic had a mean CAQ score of 38.94 (SD = 7.89), and a negative overall impression of chiropractic had a mean CAQ score of 21.19 (SD = 10.98).

**Table 2:**
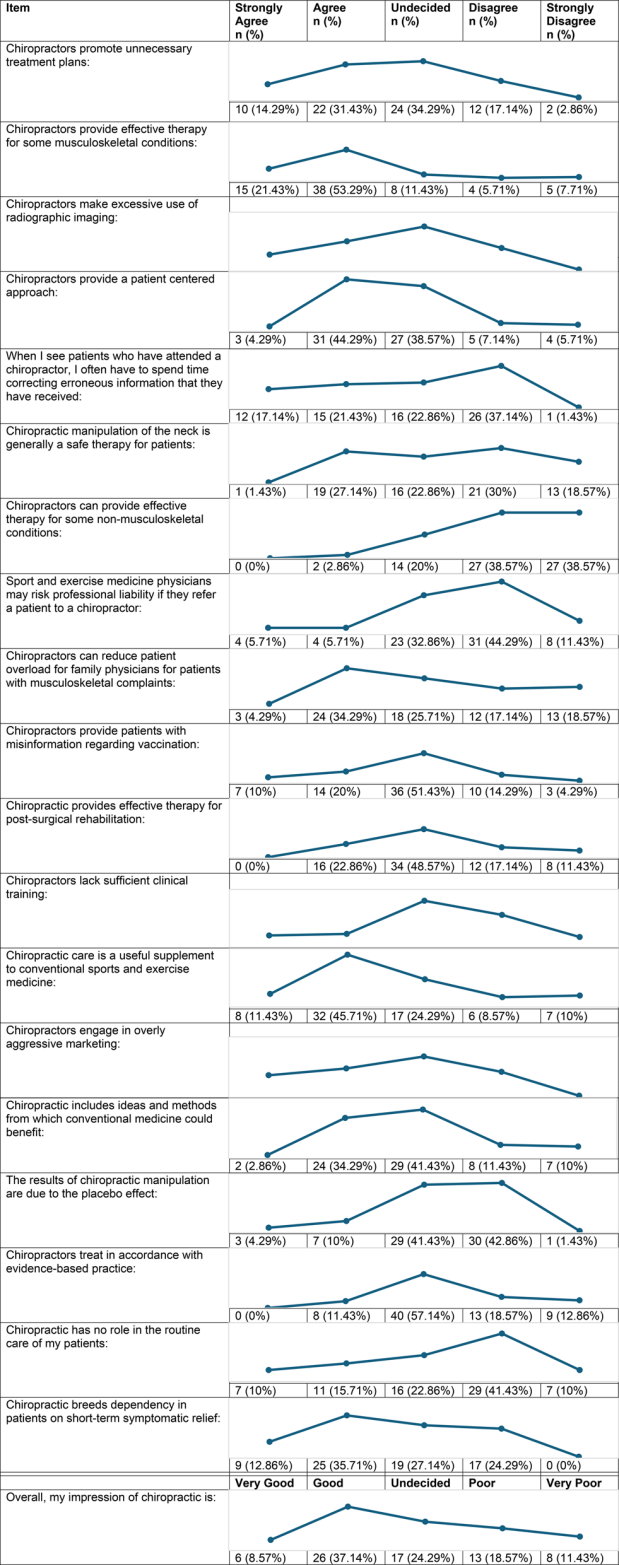
Responses to the chiropractic attitude questionnaire items (n = 70)

The regression analysis shows that more positive attitudes towards chiropractic were associated with the number of patients referred for chiropractic care in a typical year (4.97 points on the 80-point CAQ; 95% CI 2.83 to 7.11) and with a sport and exercise medicine residency that included favourable information about chiropractic (9.07 points on the 80-point CAQ; 95% CI 0.86 to 17.29). While more negative attitudes were associated with the belief that adverse events are common with chiropractic care (− 5.79 points on the 80-point CAQ; CI − 8.21 to − 3.37), and with research literature as an information source for chiropractic (− 9.06 points on the 80-point CAQ; 95% CI − 14.89 to − 3.23). The regression explains approximately 62% of the variation (adjusted R^2^ = 0.62) in Canadian sport and exercise medicine physicians’ attitudes towards chiropractic (see Table).

#### Hypothesis 2

Those CSPs who currently work with or have worked with a chiropractor in various sports medicine settings hold a more positive opinion of chiropractic, as indicated by a higher CAQ score. The independent-samples t-tests revealed that working with a chiropractor at a multisport games (i.e. Olympics, Pan Am Games, etc.) was associated with an increased CAQ score: (Yes, M = 46.15, SD = 9.08, No, M = 36.18, SD = 16.42); t(60.83) =  − 3.23, *p* < 0.01, as was previous experience working with a chiropractor in a community-based clinic (Yes, M = 45.36, SD = 10.58, No, M = 35.51, SD = 16.48); t(66.45) =  − 3.04, *p* < 0.01. However, working with a chiropractor as members of a specific IST with a NSO was not significant (Yes, M = 38.56, SD = 15.26, No, M = 39.17, SD = 15.47); t(68) = 0.14, *p* = 0.89.

Additionally, the ANOVA, with the three settings combined, found a statistically significant relationship between experience with a chiropractor in a private community-based clinic or as members of a specific Integrated Support Team with a National Sport Organization or as part of the medical team at a multisport games and a more positive CAQ score: F(4, 65) = 4.94, *p* < 0.05. Post hoc tests using the Bonferroni correction revealed there were significant differences in CAQ score between ‘Integrated Support Team’ and ‘multisport games’ (*p* = 0.02), ‘community based’ (*p* = 0.00), all three settings (*p* = 0.00), and none of the settings (*p* = 0.04). No other pairwise comparisons were significant.

#### Hypothesis 3

When looking at the relationship between having a favourable relationship with a specific chiropractor and opinion of chiropractic an independent-samples t-test indicates that there is a significant difference between CAQ scores for CSPs who work or have worked with a chiropractor in a multidisciplinary setting (M = 42.03, SD = 14.99) and for CSPs who have not (M = 29.41, SD = 13.10); t(58) = 3.27, *p* < 0.01.

An independent-samples t-test indicates that there is a significant difference between CAQ scores for those who reported that a relationship with a specific chiropractor drives their referrals (M = 44.96, SD = 11.47) and for those who do not (M = 35.52, SD = 16.316); t (65.83) = 2.83, *p* < 0.01. This suggests that those CSPs whose referrals are driven by a relationship with a specific chiropractor have a more positive opinion of chiropractic compared to those who do not.

#### Hypothesis 4

An independent-samples t-test indicated that there was no significant difference between CAQ scores for those who had exposure to chiropractic during their sport and exercise medicine residency programme (M = 39.05, SD = 16.79) and those without (M = 39.00, SD = 13.38); t (68) = 0.01, *p* = 0.99. However, an independent-samples t-test indicates that there is a significant difference between CAQ scores of CSPs who reported attending the University of Toronto for their sport and exercise medicine residency (M = 46.75, SD = 8.66) and CSPs who reported attending other institutions for their residency (M = 37.43, SD = 15.95); t (29.11), *p* < 0.01. This suggests that University of Toronto graduates have a more positive opinion of chiropractic compared to graduates from other programmes.

## Discussion

Our survey of CSPs found that their attitudes towards chiropractic and its use for the treatment of ACSE was overall positive (45.71%) or neutral (24.29%) versus negative (30.00%).

When compared to family physicians and obstetricians the mean CAQ is almost identical—39.03 compared to 40.45, 41.70 and 41.20. CSPs do have a slightly more favourable view toward chiropractic than orthopedic surgeons—39.03 compared to 34.70 (see Table 3). Comparing the responses from CSPs to the three previously surveyed groups shows similarities in agreement that chiropractors provide effective therapy for some musculoskeletal conditions and the sentiment that in general, manipulation of the neck is not a safe procedure. Regarding their overall impression of chiropractic, only 30% of Canadian obstetricians and 29% of orthopedic surgeons expressed a favourable opinion compared to 49% of Canadian family physicians and 45% of CSPs.

**Table 3:** CAQ scores compared between medical specialties

Data	Sample	Mean	Std Dev	Max	Min	Range	Overall positive view	Overall neutral view	Overall negative view
CSP	70	39.03	15.32	68	0	68	45.71%	24.29%	30.00%
North American Orthopedic Surgeons (Busse et al., 2009)	487	34.70	11.90	68	4	62	29.40%	26.10%	44.50%
Canadian Family Physicians (Busse et al., 2021)	251 (2010) 162 (2019)	40.45 41.70	12.18 13.86	73 72	0 8	73 64	47% 49%	27% 27%	26% 24%
Obstetricians (Weis et al., 2015)	91	41.20	11.70	69	0	69	30%	37%	33%

The authors felt their results suggested that medical doctors perceive the treatment of sports injuries and rehabilitation as a legitimate sector of activity for chiropractors [9–11, 13]. In our sample more than half (54.29%) of respondents worked with or had worked with a chiropractor with ‘in a community-based multidisciplinary clinic’ being the most common scenario. This formed the basis for our hypothesis that CSPs who worked with or had worked with a DC in the various sports medicine scenarios previously listed would have a more positive attitude towards chiropractic. The results demonstrated that CSPs that reported to have worked with a chiropractor in a community-based clinic or at a multisport games had a more positive attitude towards chiropractors than those who had not.

The collaborative nature of some sports medicine scenarios provide a setting where common ground shared by team members fosters collaborative activity that can lead to a better understanding of the role for chiropractors. The responses revealed that 68.57% of CSPs felt they were moderately or highly knowledgeable about chiropractic and more than half of them (55.71%) had received chiropractic care. The majority (68.7%) refer patients to chiropractors with ‘patient request’ and reported a ‘relationship with a specific chiropractor’ driving the referrals. Our results showed that CSPs who reported that a relationship with a specific chiropractor drove their referrals had more positive attitude towards chiropractors than those that did not. Very few (4.29%) respondents had received favorable information about chiropractic prior to their sports residency training. Slightly more (11.43%) received favourable information about chiropractic during their sports residency but just under half (42.86%) did not receive any information at all. See Table 4 for details on CSP’s sources of information about chiropractic and referral practices. We found it interesting that CSPs who completed their sport and exercise medicine residency at the University of Toronto had a more positive attitude towards chiropractic than those that did not. More than half of respondents (55.71%) reported that they were interested in learning more about chiropractic and nearly two thirds (62.86%) felt that their training should ‘definitely’ include information about chiropractic. Only 12.86% reported that chiropractic should not be made available in high performance settings. The vast majority (90%) agreed that variability in the chiropractic profession is a barrier to greater collaboration with CSPs (Table 5).

**Table 4:** CSPs sources of information on chiropractic and referral practices

Received chiropractic treatment as a patient, n (%)		
Yes	39 (55.71%)	
No	31 (44.29%)	
Self-rated knowledge of chiropractic, n (%)		
No knowledge	1 (1.43%)	
A little knowledgeable	21 (30%)	
Moderately knowledgeable	34 (48.57%)	
Very knowledgeable	14 (20%)	
Did your medical training, prior to your sport and exercise medicine residency, expose you to information about chiropractic? n (%)		
Yes, overall favourable	3 (4.29%)	
Yes, overall neutral	14 (20%)	
Yes, overall unfavourable	21 (30%)	
No	32 (45.71%)	
Did your sport and exercise medicine residency expose you to information about chiropractic? n (%)		
Yes, overall favourable	8 (11.43%)	
Yes, overall neutral	25 (35.71%)	
Yes, overall unfavourable	7 (10%)	
No	30 (42.86%)	
How has your opinion of chiropractic been formed? n (%)		
Personal experience as a patient	31 (44.29%)	
Patient feedback	51 (72.86%)	
Family and friends	21 (30%)	
Professors/supervisors/mentors	30 (42.86%)	
Research literature	21 (30%)	
Relationship with a specific chiropractor	37 (52.86%)	
Media	2 (2.86%)	
Residency	22 (31.43%)	
Medical school	14 (20%)	
Work experience (using chiropractic as a treatment for patients)	43 (61.43%)	
I have no opinion on chiropractic	1 (1.43%)	
Other	4 (5.71%)	
When were your opinions of chiropractic predominantly formed? n (%)		
Before medical school	12 (17.14%)	
During medical school	11 (15.71%)	
After medical school	45 (64.29%)	
I have no opinion on chiropractic	2 (2.86%)	
How many patients do you refer for chiropractic care in a typical year? n (%)		
None	22 (31.43%)	
1–10	22 (31.43%)	
11–25	12 (17.14%)	
26–50	7 (10%)	
More than 50	7 (10%)	
If you do refer patients for chiropractic care, what drives the referrals? n (%)		
Patient request	38 (54.29%)	
Non-response to medical treatment	18 (25.71%)	
Literature supports chiropractic care for certain conditions	18 (25.71%)	
Relationship with a specific chiropractor	26 (37.14%)	
My own positive experience as a chiropractic patient 5 (7.14%)		
I do not refer patients for chiropractic care	20 (28.57%)	
Other	5 (7.14%)

**Table 5:** Variables associated with Canadian sport and exercise medicine physicians’ attitudes towards chiropractic (n = 64)

Variable	Unstandardized regression coefficient from univariable analysis (95% CI)	*p*-value	Unstandardized regression coefficient from multivariable analysis (95% CI)	*p*-value
Gender	0.26 (− 7.21 to 7.73)	0.95	2.16 (− 2.99 to 7.31)	0.41
Received chiropractic care	− 0.57 (− 7.98 to 6.84)	0.88	3.03 (− 2.48 to 8.53)	0.28
Number of patients referred for chiropractic care in a typical year	6.34 (3.93 to 8.75)	0.00	4.97 (2.83 to 7.11)	0.00
Information source for chiropractic^a^				
Professors/supervisors/mentors	− 1.74 (− 9.17 to 5.68)	0.64	5.13 (− 0.71 to 10.96)	0.08
Research Literature	− 15.01 (− 22.17 to -7.84)	0.00	− 9.06 (− 14.89 to − 3.23)	0.00
Media	− 21.65 (− 43.11 to -0.19)	0.05	− 10.61 (− 26.78 to 5.56)	0.19
Residency	− 6.01 (− 13.80 to 1.79)	0.13	− 4.29 (− 10.50 to 1.92)	0.17
Work experience (using chiropractic patient treatment)	6.68 (− 0.71 to 14.06)	0.08	− 4.72 (− 10.81 to 1.38)	0.13
Sport and exercise medicine residency included favourable information about chiropractic	14.93 (3.94 to 25.92)	0.01	9.07 (0.86 to 17.29)	0.03
Belief that adverse events are common with chiropractic care	− 7.63 (− 10.37 to − 4.89)	0.00	− 5.79 (− 8.20 to − 3.37)	0.00

95% CI = 95% confidence interval

a = each sub-category was entered individually into the regression model as respondents could endorse multiple categories

The results of our survey suggest that CSPs who have had more exposure to chiropractors had more positive attitudes towards chiropractic treatment for ACSE than those who had not. Further integration of chiropractors into multidisciplinary sports medicine teams over time should serve to enhance the progress that has been made in this regard. The inclusion of a clinical placement with a chiropractor is a relatively new development but the results suggest that those that had this exposure had a higher CAQ score. The responses suggest that there is an interest amongst most CSPs to learn more about chiropractic.

### Strengths and limitations

Strengths of our study include distribution of the survey to all active physician members of CASEM via their monthly newsletter in addition to targeting those members attending the annual symposium in 2023. Due to the nature of the Survey Monkey software we were able to limit responses to one per browser or email address however it remained possible that a motivated responder could have circumvented this by clearing cookies on their browser or take the survey again on a different browser or device. Our analysis of the time stamps related to each completed survey suggests that there were no serial responders who responded more than once.

The CAQ has been applied and validated among other groups of healthcare providers. Non-responders may be systematically different to our participants, and our results may have limited generalizability both beyond our sample and outside of Canada. While the response rate (11%) makes the generalizability of our results uncertain, it is similar to the response rates for Canadian family physicians in 2019 (7%) and Canadian obstetricians (14%) [10, 11]. The response rates for Canadian family physicians in 2010 was 37% and North American orthopaedic surgeons was 49%. [9, 10]

Due to the small sample size, we were also unable to perform regression analyses for hypotheses 2–4 and were therefore unable to assess the impact of additional independent variables on the relationship with CAQ scores. When regressions were explored for these hypotheses, they were all underpowered and lacking in significance, hence the use of t-tests and the ANOVAs to explore these relationships.

## Conclusion

CSPs attitudes towards chiropractors are diverse but overall positive. Our results provide evidence that previous recommendations to increase opportunities for medical doctors and chiropractors to interact can improve relations as CSPs who reported to have worked with a chiropractor in various sports medicine settings, including exposure to chiropractic during their sports and exercise medicine training, have more positive attitudes than those who have not.

## Electronic supplementary material

Below is the link to the electronic supplementary material.

Supplementary Material 1

Supplementary Material 2

Supplementary Material 3

## Data Availability

The data and analyses is available from the corresponding author on reasonable request.
