# Supplementary material for: Attitudes towards chiropractic: a survey of Canadian sport and exercise medicine physicians
Source: Chiropr Man Therap. 2025 May 20;33:19. doi: 10.1186/s12998-025-00581-4 (PMC12093891; doi:10.1186/s12998-025-00581-4)
Supplement: Supplementary file 1 — Supplementary Material 1 [file 12998_2025_581_MOESM1_ESM.docx]

**APPENDIX A**

Chiropractic Attitude Questionnaire (CAQ) - Attitudes towards chiropractic: a survey of Canadian Sport and Exercise Medicine Physicians.

1. What is your gender identity?

☐ Male

☐ Female

☐ Other

1. Type of practice (**please check all that apply**):

□ Community

□ Hospital-based

□ Multidisciplinary

□ Private practice

□ Academic

□ Other: _______________________________________________

3. Do you currently work with, or have you worked with, a chiropractor in a multidisciplinary setting?

- Yes
- No
- No, but I would be open to working with a chiropractor
- Unsure
- I do not work in a multidisciplinary setting

4. If yes, in which setting have you worked with a chiropractor? (please check all that apply):

☐ Integrated Support Team (National Sport Organization)

☐ Professional Sports Team

☐ At a multisport games (i.e. Olympics, Pan Am Games, Commonwealth, etc.)

☐ At a single sport competition (i.e. Provincial, National, International competition, etc.)

☐ In a community based sports medicine clinic

☐ Other:

☐ Not Applicable

5. What best describes the proportion of your patients that have musculoskeletal conditions?

☐ 0-25%

☐26-50%

☐ 51-75%

☐76-100%

6. What best describes your area(s) of clinical interest?

□ General family medicine □ Occupational medicine

□ Emergency medicine □ Sports medicine

□ Psychotherapy/Psychiatry □ Pediatrics

□ Pain medicine ☐ Orthopaedics

☐ Physiatry

□ Other ____________________________________________________

7. Years in practice: □ Less than 5 years

□ 5 to 10 years

□ 11 to 20 years

□ More than 20 years

8. How would you rate your knowledge of chiropractic?

□ I have no knowledge

□ A little knowledgeable

□ Moderately knowledgeable

□ Very knowledgeable

9. Have you ever received chiropractic care?

☐ Yes

☐ No

10. Did your medical training, prior to your sport and exercise medicine residency, expose you to information about chiropractic?

- Yes, and the information was overall favorable
- Yes, and the information was overall neutral
- Yes, and the information was overall unfavorable
- No

11. At which institution did you complete your sport and exercise medicine residency training?

12. Did your sport and exercise medicine residency program expose you to information about chiropractic?

☐ Yes, and the information was overall favorable

☐ Yes, and the information was overall neutral

☐ Yes, and the information was overall unfavorable

☐ No

13. Should sports and exercise medicine training include exposure to information about chiropractic?

- Yes, definitely
- Possibly

□ Unsure

□ Probably not

□ No, definitely not

14. Do you feel comfortable discussing chiropractic care with your patients?

- Yes, definitely
- Somewhat

□ Unsure

□ Probably not

□ No, definitely not

15. Are you interested in learning more about chiropractic?

- Yes
- No
- Unsure

16. Do you have any training in spinal manipulation?

- Yes, and I use this modality with some of my patients
- Yes, but I do not use this modality with my patients
- No

17. Would you like to receive training in spinal manipulation?

- Yes
- No
- Unsure

18. How has your opinion of chiropractic been formed? (**Please check all that apply**)

□ Personal treatment experience □ Patient feedback

□ Family and friends □ Professors/supervisors/mentors

□ Research Literature □ Relationship with a chiropractor

□ Media □ Residency

□ Medical school □ Work experience

☐ Other _________________

□ I have no opinion on chiropractic

19. When were your opinions of chiropractic predominantly formed?

□ Before medical school

□ During medical school

□ After medical school

20. Should chiropractic care be available in “high performance” settings (i.e. National Sport Organizations, multisport games, professional sports teams, etc.)

☐ Yes

☐ No

☐ Unsure

21. How frequently do you refer your patients for chiropractic care?

□ Daily

□ Weekly

□ Monthly

□ Every year

□ Never

22. How many patients do you refer for chiropractic care in a typical year?

□ None

□ 1 to 10

□ 11 to 25

□ 26 to 50

□ More than 50

23. If you do refer patients for chiropractic care, what drives your referrals?

(**Please check all that apply**)

□ Patient request

- Non-response to medical treatment
- Literature supports chiropractic care for certain conditions
- Relationship with specific chiropractor
- My own positive experience as a chiropractic patient
- I do not refer patients for chiropractic care
- Other_____________________________________________________

24. Does diversity within the chiropractic profession present a barrier to greater collaboration with sport and exercise medicine physicians?

- Yes
- No
- Unsure

25. Do you perceive chiropractors as primary care providers?

- Yes, definitely
- Somewhat

□ Unsure

□ Probably not

□ No, definitely not

26. Would you like to receive a consultation note from a chiropractor who has seen one of your patients?

- Yes
- No
- Unsure
- Not applicable in my practice setting

27. Have patients been referred to your practice by chiropractors in order for you to refer them for imaging studies?

- Yes
- No
- Not applicable in my practice setting

28. Do you perceive that adverse events associated with chiropractic care are common?

- Yes
- Yes, but serious adverse events are rare
- No
- Unsure

29. Are you aware that chiropractors require a minimum of three years of undergraduate university level education prior to a four-year chiropractic education?

Yes

No

30. Chiropractors promote unnecessary treatment plans:

Strongly Agree □ Agree □ Undecided □ Disagree □ Strongly Disagree □

31. Chiropractors provide effective therapy for some musculoskeletal conditions:

Strongly Agree □ Agree □ Undecided □ Disagree □ Strongly Disagree □

32. Chiropractors make excessive use of radiographic imaging:

Strongly Agree □ Agree □ Undecided □ Disagree □ Strongly Disagree □

33. Chiropractors provide a patient centered approach:

Strongly Agree □ Agree □ Undecided □ Disagree □ Strongly Disagree □

34. When I see patients who have attended a chiropractor, I often have to spend time correcting erroneous information that they have received:

Strongly Agree □ Agree □ Undecided □ Disagree □ Strongly Disagree □

35. Chiropractic manipulation of the neck is generally a safe therapy for patients:

Strongly Agree □ Agree □ Undecided □ Disagree □ Strongly Disagree □

36. Chiropractors can provide effective therapy for some non-musculoskeletal conditions (e.g. asthma, infantile colic):

Strongly Agree □ Agree □ Undecided □ Disagree □ Strongly Disagree □

37. Sport and exercise medicine physicians may risk professional liability if they refer a patient to a chiropractor:

Strongly Agree □ Agree □ Undecided □ Disagree □ Strongly Disagree □

38. Chiropractors can reduce patient overload for family physicians for patients with musculoskeletal complaints:

Strongly Agree □ Agree □ Undecided □ Disagree □ Strongly Disagree □

39. Chiropractors provide patients with misinformation regarding vaccination:

Strongly Agree □ Agree □ Undecided □ Disagree □ Strongly Disagree □

40. Chiropractic provides effective therapy for post-surgical rehabilitation:

Strongly Agree □ Agree □ Undecided □ Disagree □ Strongly Disagree □

41. Chiropractors lack sufficient clinical training:

Strongly Agree □ Agree □ Undecided □ Disagree □ Strongly Disagree □

42. Chiropractic care is a useful supplement to conventional sports and exercise medicine:

Strongly Agree □ Agree □ Undecided □ Disagree □ Strongly Disagree □

43. Chiropractors engage in overly aggressive marketing:

Strongly Agree □ Agree □ Undecided □ Disagree □ Strongly Disagree □

44. Chiropractic includes ideas and methods from which conventional medicine could benefit:

Strongly Agree □ Agree □ Undecided □ Disagree □ Strongly Disagree □

45. The results of chiropractic manipulation are due to the placebo effect:

Strongly Agree □ Agree □ Undecided □ Disagree □ Strongly Disagree □

46. Chiropractors treat in accordance with evidence-based practice:

Strongly Agree □ Agree □ Undecided □ Disagree □ Strongly Disagree □

47. Chiropractic has no role in the routine care of my patients:

Strongly Agree □ Agree □ Undecided □ Disagree □ Strongly Disagree □

48. Chiropractic breeds dependency in patients on short-term symptomatic relief:

Strongly Agree □ Agree □ Undecided □ Disagree □ Strongly Disagree □

49. Overall, my impression of chiropractic is:

Very Good □ Good □ Undecided □ Poor □ Very Poor □

Please share with us any other thoughts you may have on chiropractic.

**- Thank You for Your Time**
