# Supplementary material for: Attitudes towards chiropractic: a survey of Canadian sport and exercise medicine physicians
Source: Chiropr Man Therap. 2025 May 20;33:19. doi: 10.1186/s12998-025-00581-4 (PMC12093891; doi:10.1186/s12998-025-00581-4)
Supplement: Supplementary file 2 — Supplementary Material 2 [file 12998_2025_581_MOESM2_ESM.docx]

**Manuscript Title**: Attitudes towards chiropractic: a survey of Canadian sport and exercise medicine physicians

**Authors**: Cameron Borody DC FRCCSS(C) Canadian Memorial Chiropractic College, Janet D’Arcy DC FRCCSS(C) Canadian Memorial Chiropractic College, Jaime Waters PhD The Open University, Mark Leung MD University of Toronto, Jason Busse PhD McMaster University

**Correspondence**: Dr. Cameron Borody, Canadian Memorial Chiropractic College, 6100 Leslie St, Toronto ON, M2H 3J1. Phone: 416-482-2340 Fax: 416-646-1115 Email: [cborody@cmcc.ca](mailto:cborody@cmcc.ca)

**Word Count:** Abstract (372), Manuscript (3,294)

**Background**: Previous surveys of physicians revealed diverse attitudes towards chiropractic. One of several strategies proposed by leaders in chiropractic to support the advancement of the profession in North America is to increase opportunities for interprofessional collaboration. One area where chiropractic has achieved some degree of integration in Canada is in the field of sports medicine. A result of the increased integration of chiropractic in the field of sports medicine has been more opportunity for interprofessional collaboration, development of

interprofessional relationships and increased exposure to chiropractic. The purpose of this study is to determine the attitudes and their contributing factors of Canadian sport and exercise medicine physicians (CSPs) toward chiropractic and its use for treatment of athletes and/or Canadians who are participating in sports or exercise (ACSE) We will compare our results with previously studied populations.

**Methods:** An invitation to complete the survey was included in a newsletter emailed to active physician members of CASEM (Canadian Academy of Sports and Exercise Medicine) in March and April 2023 and attendees of their symposium. The survey included the Chiropractic Attitude Questionnaire (CAQ), which allowed respondents to indicate their attitudes towards chiropractic care for ACSE using a 5-point Likert scale. The responses to the CAQ were the primary outcome measure. Descriptive statistics, including mean, median, standard deviation, maximum, minimum and range, regression analysis, t-tests, and ANOVAs were used to analyse the data.

**Results**: Seventy CSPs completed the survey (response rate: 11%). The summed CAQ scores ranged from 0 to 68 with a mean of 39.03 and a standard deviation of 15.30. Compared to family physicians (2010 and 2019) and obstetricians (2016) who completed the survey the mean CAQ is almost identical – 39.03 compared to 40.45, 41.70 and 41.20. CSPs who worked with a chiropractor in a multidisciplinary setting hold a more positive opinion of chiropractic. An independent samples t-test indicated that there was a significant difference between CAQ scores for those with experience of working with a chiropractor (M=42.03, SD=14.99) and those without (M=29.41, SD=13.10); t(58) = 3.27, p < 0.05.

**Conclusions**: CSPs attitudes toward chiropractic and its use for treatment of ACSE were similar to those of previously surveyed medical specialties. CSPs who reported to have worked with a chiropractor have more positive attitudes than those that have not.

**Key words**: chiropractic, attitude, sports medicine, survey

**Background**

Previous work that applied the Chiropractic Awareness Questionnaire (CAQ) in surveys of North American orthopedic surgeons, Canadian family physicians and Canadian obstetricians revealed diverse attitudes towards chiropractic.^1-4^ (See Table 3) In 2006, a strategic planning conference for the chiropractic profession made several recommendations including prioritizing the development and implementation of a strategy to increase interprofessional collaboration.^6^ There is some evidence of progress in this regard with the inclusion of chiropractors in some Family Health Teams, working as Advanced Practice Practitioners screening patients for spine surgeons and the integration of chiropractic into some private sector medical facilities.^7-11^ In the pursuit of excellence in athletic performance, sports medicine has evolved to embrace an interdisciplinary model of care commonly referred to in Canada as an Integrated Support Team (IST) that often includes a chiropractor.^12^ Interviews of stakeholders on sports medicine healthcare teams has suggested chiropractors’ inclusion was a manifestation of ‘consumer-focused practice’ and that ‘the athlete’s wanted them’ not necessarily that the profession had been accepted by other healthcare professionals.^13^ Nonetheless, chiropractors have continued to integrate into the field of sports medicine. In 2010, at the Winter Olympics in Vancouver, chiropractic was included for the first time as an equal partner in the delivery of healthcare to all participants. It is now very common in Canada, particularly in urban centres, for private multidisciplinary clinics marketing themselves as “sports medicine” to include a chiropractor. The sports and exercise medicine residency programme at the University of Toronto has included a clinical placement with a chiropractor for its residents since 2014. At the 2023 Pan American Games in Chile, the Canadian Medical Services Team included 53 health care providers, of which five were chiropractors. In Canada, the sports medicine landscape has allowed for various opportunities for chiropractors to collaborate with Canadian sport and exercise medicine physicians (CSPs). Understanding how CSPs view chiropractic may provide information to examine the impact of improved integration, opportunities to enhance interprofessional collaboration and ultimately improve patient care. This study aims to evaluate the attitudes (hypothesis 1), and their contributing factors (hypotheses 2-4), of CSPs towards chiropractic. We will also compare our results with previously studied populations (hypothesis 1).

**Methods:**

We adapted a questionnaire previously used to evaluate attitudes and beliefs of orthopedic surgeons towards chiropractic for our study.^1^ Specific modifications include more details pertaining to medical training and practice experience resulting in a 49-item questionnaire that examined the attitudes of CSPs towards chiropractic care of athletes and/or Canadians who participate in sports or exercise (ACSE) (Appendix-A). The questionnaire included a 20-item section, the Chiropractic Attitude Questionnaire (CAQ), which captured respondent’s attitudes towards chiropractic. The remaining 29 items related to demographics and other professional characteristics and activities. The clinimetric properties of the CAQ were established in previous studies as part of its original development.^1-3^ Respondents were invited to enter written comments in a final open-ended component at the end of the survey.

The survey was reviewed independently by ML and two residents in the enhanced skills sports and exercise medicine residency programme at the University of Toronto for suitability and relevance to their profession.

**Subjects**: The inclusion criteria of this study consisted of medical doctors who are licensed and active members of the Canadian Academy of Sport and Exercise Medicine (CASEM) in any Canadian province or territory.

**Procedures**: Respondents were CSPs that received the survey through distribution to the CASEM database and/or attended the 2023 CASEM symposium. We approached CASEM and they agreed to include an invitation to participate in our survey in their monthly newsletter. The invitation included a link to the online survey where participants were presented with a disclosure letter and the questionnaire. We used Survey Monkey to facilitate online completion of the questionnaire. The study disclosure letter notified potential participants of the purpose of the study, what their involvement would entail, and the risks and benefits of partaking in the study, as well as a disclaimer notifying them that they were not obligated to participate. If a potential participant agreed to the terms of the study they indicated as such by clicking on an “I agree” button that linked them to the survey. Only actively practicing CSPs were asked to complete the survey.

CASEM included the invitation to participate in our study in their electronic newsletter twice. It was sent to all 1045 CASEM members in March and April of 2023. At that time their membership included 638 active CSPs, which was our target population. CASEM provided us with some analytics from the Mail Chimp online distribution platform showing that in March and April, 289 and 280 recipients of the email clicked on the link to the newsletter that contained our invitation.

CASEM also agreed to include a card with a QR code linking to our survey in the lanyards for the 381 CSPs who attended the 2023 CASEM symposium in Banff, Alberta. The survey was available to potential respondents for three months.

There was no identifying information to link the respondent to a specific questionnaire and due to the nature of the software respondents were only able to complete the questionnaire once. Because Survey Monkey takes the IP addresses of respondents a research assistant stripped that information from the completed surveys prior to the research team reviewing the data. Respondents were asked if they were interested in the survey results and were prompted to provide their email address so that a summary of the study could be distributed to them upon completion of the study~~.~~

There was no compensation for participating in the study however respondents were asked if they would like to submit their email address to be included in a random draw for one of five $50 gift cards. A research assistant removed the email addresses from the surveys of those that submitted for the random draw and after the data collection period was completed a randomization software programme was used to identify five email addresses to award a gift card.

**Outcome Measures**: The primary outcome measure for this study was the CSP’s attitudes towards chiropractic and its use for treatment of ACSE as determined by the total score on the CAQ.

**Analysis**: Each of the 20 questions comprising the CAQ was graded on a 5-point Likert scale, from 0 to 4. The responses were then summed to arrive at a total score ranging from 0 (most negative attitude towards chiropractic) to 80 (most positive attitude towards chiropractic). Demographic frequencies for gender identity, years in practice, and previous work experience were calculated. For the evaluation of the hypotheses listed below a statistical test was considered statistically significant if p < .05. Written comments provided by respondents will be evaluated in a separate analysis.

Hypothesis (1): CSPs will hold a more positive opinion toward chiropractic than previously published data from other surveyed medical specialties. We applied a qualitative comparison of our CAQ scores out of 80 with previously published data. We conducted an inspection of the CAQ scores including mean, median, standard deviation, maximum, minimum and range and compared them to previously published data. We also carried out a regression analysis to explore the factors influencing attitudes towards chiropractic.

Hypothesis (2): CSPs who currently work with or have worked with a chiropractor in a private community-based clinic or as members of a specific IST with a National Sport Organization (NSO) or as part of the medical team at a multisport games will hold a more positive opinion of chiropractic. We tested this hypothesis in two ways. First, we combined all responses that confirmed previous work experience with a chiropractor in any of the specific scenarios detailed above and applied a t-test to investigate the relationship between previous experience working with a chiropractor (group/independent variable) and the CAQ score (test/dependent variable). Secondly, we applied an ANOVA test where each of the three different scenarios where respondents have previous experience working with a chiropractor or no previous experience working with a chiropractor to investigate the relationship between the different work experience scenarios (group/independent variable) and the CAQ score (test/dependent variable).

Hypothesis (3): CSPs indicating a favourable relationship with a specific chiropractor will hold a more positive opinion toward chiropractic. We applied a t-test to investigate the relationship between CSPs who indicated a favourable relationship with a specific chiropractor (grouping/independent variable) and the CAQ score (test/dependent variable).

Hypothesis (4): CSPs who indicate they had exposure to chiropractic during their sport and exercise medicine residency programme will hold a more positive opinion toward chiropractic. We applied an ANOVA test to investigate the relationship between the different exposure scenarios (Yes, overall favourable, yes, overall neutral, yes, overall unfavourable and no exposure) and the CAQ score.

**Results:**

The link to the survey was sent to all 638 active physician members of CASEM. Eighty-two recipients responded and 70 completed the survey (response rate: 11%). Despite the low response rate and self-selecting nature of participation, there remains a certain amount of generalisability of the findings due to the inclusion of the entire population in the sampling frame.

**Demographics:** Participant demographics were examined in terms of gender identity, years in practice, and previous work experience (see Table-1). Over half of CSPs (38 of 70, 54.29%) had worked with a chiropractor in a multidisciplinary setting, and over two-thirds (48 of 70, 68.57%) had referred at least one patient to a chiropractor in the last year.

**Attitudes towards Chiropractic:** Just under half (45.71%) of CSPs had an overall positive impression of chiropractic, compared to 30.00% who had a negative view, and 24.29% whose overall impression was neutral. Individual responses for each of the twenty questions can be found in Table-2. Most questions did not elicit a particularly strong responses one way or the other, with the exception of ‘Chiropractors provide effective therapy for some musculoskeletal conditions’ where the majority (75.71%) of CSPs agreed / strongly agreed and ‘Chiropractors can provide effective therapy for some non-musculoskeletal conditions (e.g. asthma, infantile colic), where the majority (77.14%) of CSPs disagreed / strongly disagreed. Overall, the summed CAQ score ranged from 0 to 68 with a mean of 39.03 and a standard deviation of 15.32. Breaking this down, those with a positive overall impression of chiropractic had a mean CAQ score of 50.78 (SD = 7.36), while an undecided overall impression of chiropractic had a mean CAQ score of 38.94 (SD = 7.89), and a negative overall impression of chiropractic had a mean CAQ score of 21.19 (SD = 10.98).

**Hypothesis 1:** CSPs hold a similar opinion toward chiropractic than previously published data from surveyed medical specialties (Table-3). When compared to family physicians and obstetricians the mean CAQ is almost identical – 39.03 compared to 40.45, 41.70 and 41.20. CSPs do have a slightly more favourable view toward chiropractic than orthopedic surgeons – 39.03 compared to 34.70.

A regression analysis shows that more positive attitudes towards chiropractic were associated with the number of patients referred for chiropractic care in a typical year (4.97 points; 95% CI 2.83 to 7.11) and with a sport and exercise medicine residency that included favourable information about chiropractic (9.07 points; 95% CI 0.86 to 17.29). While more negative attitudes were associated with the belief that adverse events are common with chiropractic care (-5.79 (-8.21 to -3.37), and with research literature as an information source for chiropractic (-9.06 points; 95% CI -14.89 to -3.23). The regression explains approximately 62% of the variation (adjusted R^2^ = 0.62) in Canadian sport and exercise medicine physicians’ attitudes towards chiropractic (see Table-5).

**Hypothesis 2:** Those CSPs who currently work with or have worked with a chiropractor in various sports medicine settings hold a more positive opinion of chiropractic, as indicated by a higher CAQ score. The independent-samples t-tests revealed that working with a chiropractor at a multisport games (i.e. Olympics, Pan Am Games, etc.) was associated with an increased CAQ score: (Yes, M=46.15, SD=9.08, No, M= 36.18, SD = 16.42); t(60.83) = -3.23, p < 0.01, as was previous experience working with a chiropractor in a community-based clinic (Yes, M = 45.36, SD = 10.58, No, M = 35.51, SD = 16.48); t(66.45) = -3.04, p < 0.01. However, working with a chiropractor as members of a specific IST with a NSO was not significant (Yes, M = 38.56, SD = 15.26, No, M = 39.17, SD = 15.47); t(68) = 0.14, p = 0.89.

Additionally, the ANOVA, with the three settings combined, found a statistically significant relationship between experience with a chiropractor in a private community-based clinic or as members of a specific Integrated Support Team with a National Sport Organization or as part of the medical team at a multisport games and a more positive CAQ score: F(4, 65) = 4.94, p<0.05. Post hoc tests using the Bonferroni correction revealed there were significant differences in CAQ score between ‘Integrated Support Team’ and ‘multisport games’ (p=0.02), ‘community based’ (p=0.00), all three settings (p=0.00), and none of the settings (p=0.04). No other pairwise comparisons were significant.

**Hypothesis 3:** When looking at the relationship between having a favourable relationship with a specific chiropractor and opinion of chiropractic an independent-samples t-test indicates that there is a significant difference between CAQ scores for CSPs who work or have worked with a chiropractor in a multidisciplinary setting (M=42.03, SD=14.99) and for CSPs who have not (M=29.41, SD=13.10); t(58) = 3.27, p < 0.01.

An independent-samples t-test indicates that there is a significant difference between CAQ scores for those who reported that a relationship with a specific chiropractor drives their referrals (M = 44.96, SD = 11.47) and for those who do not (M = 35.52, SD = 16.316); t (65.83) = 2.83, p < 0.01. This suggests that those CSPs whose referrals are driven by a relationship with a specific chiropractor have a more positive opinion of chiropractic compared to those who do not.

**Hypothesis 4:** An independent-samples t-test indicated that there was no significant difference between CAQ scores for those who had exposure to chiropractic during their sport and exercise medicine residency programme (M=39.05, SD=16.79) and those without (M=39.00, SD = 13.38); t (68) = 0.01, p = 0.99. However, an independent-samples t-test indicates that there is a significant difference between CAQ scores of CSPs who reported attending the University of Toronto for their sport and exercise medicine residency (M = 46.75, SD = 8.66) and CSPs who reported attending other institutions for their residency (M = 37.43, SD = 15.95); t (29.11), p < 0.01. This suggests that University of Toronto graduates have a more positive opinion of chiropractic compared to graduates from other programmes.

**Discussion**

Our survey of CSPs found that their attitudes towards chiropractic and its use for the treatment of ACSE was overall positive (45.71%) or neutral (24.29%) versus negative (30.00%).

While we expected that CSPs would have a more positive opinion towards chiropractic than previous analyses of Canadian family physicians and obstetricians the results showed that the mean CAQ scores amongst the groups were almost identical. CSPs did demonstrate a slightly more positive attitude towards chiropractors than North American orthopedic surgeons^1^.

Comparing the responses from CSPs to the three previously surveyed groups shows similarities in agreement that chiropractors provide effective therapy for some musculoskeletal conditions and the sentiment that in general, manipulation of the neck is not a safe procedure. Regarding their overall impression of chiropractic, only 30% of Canadian obstetricians and 29% of orthopedic surgeons expressed a favourable opinion compared to 49% of Canadian family physicians and 45% of CSPs. (Table-3)

The authors felt their results suggested that medical doctors perceive the treatment of sports injuries and rehabilitation as a legitimate sector of activity for chiropractors.^1-4^ In our sample more than half (54.29%) of respondents worked with or had worked with a chiropractor with ‘in a community-based multidisciplinary clinic’ being the most common scenario. This formed the basis for our hypothesis that CSPs who worked with or had worked with a DC in the various sports medicine scenarios previously listed would have a more positive attitude towards chiropractic. The results demonstrated that CSPs that reported to have worked with a chiropractor in a community-based clinic or at a multisport games had a more positive attitude towards chiropractors than those who had not.

The collaborative nature of some sports medicine scenarios provide a setting where common ground shared by team members fosters collaborative activity that can lead to a better understanding of the role for chiropractors. The responses revealed that 68.57% of CSPs felt they were moderately or highly knowledgeable about chiropractic and more than half of them (55.71%) had received chiropractic care. The majority (68.7%) refer patients to chiropractors with ‘patient request’ and reported a ‘relationship with a specific chiropractor’ driving the referrals. Our results showed that CSPs who reported that a relationship with a specific chiropractor drove their referrals had more positive attitude towards chiropractors than those that did not. Very few (4.29%) respondents had received favorable information about chiropractic prior to their sports residency training. Slightly more (11.43%) received favourable information about chiropractic during their sports residency but just under half (42.86%) did not receive any information at all. See Table-4 for details on CSP’s sources of information about chiropractic and referral practices. We found it interesting that CSPs who completed their sport and exercise medicine residency at the University of Toronto had a more positive attitude towards chiropractic than those that did not. More than half of respondents (55.71%) reported that they were interested in learning more about chiropractic and nearly two thirds (62.86%) felt that their training should ‘definitely’ include information about chiropractic. Only 12.86% reported that chiropractic should not be made available in high performance settings. The vast majority (90%) agreed that variability in the chiropractic profession is a barrier to greater collaboration with CSPs.

The results of our survey suggest that CSPs who have had more exposure to chiropractors had more positive attitudes towards chiropractic treatment for ACSE than those who had not. Further integration of chiropractors into multidisciplinary sports medicine teams over time should serve to enhance the progress that has been made in this regard. The inclusion of a clinical placement with a chiropractor is a relatively new development but the results suggest that those that had this exposure had a higher CAQ score. The responses suggest that there is an interest amongst most CSPs to learn more about chiropractic.

**Strengths and limitations**

Strengths of our study include distribution of the survey to all active physician members of CASEM via their monthly newsletter in addition to targeting those members attending the annual symposium in 2023. Due to the nature of the Survey Monkey software we were able to limit responses to one per browser or email address however it remained possible that a motivated responder could have circumvented this by clearing cookies on their browser or take the survey again on a different browser or device. Our analysis of the time stamps related to each completed survey suggests that there were no serial responders who responded more than once.

The CAQ has been applied and validated among other groups of healthcare providers. Non-responders may have differed systematically than responders, and our results may have limited generalizability outside of Canada. The response rate (11%) makes the generalizability of our results uncertain but similar to the response rates for Canadian family physicians in 2019 (7%) and Canadian obstetricians (14%).^2,3^ The response rates for Canadian family physicians in 2010 was 37% and North American orthopaedic surgeons was 49%.^1,2^

**Conclusion**

CSPs attitudes towards chiropractors are overall positive and very similar to previously surveyed medical specialties. Our results provide evidence that previous recommendations to increase opportunities for medical doctors and chiropractors to interact can improve relations as CSPs who reported to have worked with a chiropractor in various sports medicine settings, including exposure to chiropractic during their sports and exercise medicine training, have more positive attitudes than those who have not.

.

**Declarations:**

**Ethics approval and consent to participate:**

As this study was voluntary, participants were not subjected to any risk or harm. Respondents freely provided informed consent and were able to participate or withdraw at any time. The survey did not ask for any personal identification and there was no way of linking responses to a particular CSP or their email address. All surveys were stored on a secure server at the Canadian Memorial Chiropractic College.

This study was approved by the Research Ethics Board of the Canadian Memorial Chiropractic College on November 24, 2022 (certificate #2210B01).

**Consent for publication:** Not applicable.

**Availability of data and materials:**

The datasets used and/or analysed in the current study are available from the corresponding author on reasonable request.

**Competing Interests:**

The authors (CB, JD, ML, JW) declare that they have no competing interests.

JB is supported, in part, by a Canadian Institutes of Health Research Canada Research Chair in the prevention and management of chronic pain.

**Funding:**

This study was supported by a grant ($7500) from the Canadian Chiropractic Research Foundation.

**Author’s Contributions:**

CB, JD and JW prepared the grant and ethics applications. CB, JW and ML supported the data collection and JW and JB analyzed the data. CB and JW prepared the manuscript and all authors reviewed and approved the final manuscript.

**Acknowledgments:**

The authors would like to thank Dr. Carol Ann Weis, Dr. David Oh, Dawn Haworth and Mark Fillery for their support.

**References**

1. Busse JW, Jacobs C, Ngo T, et al. Attitudes toward chiropractic: A survey of North American Orthopedic Surgeons. Spine 2009;34:2818-25.
2. Busse JW, Pallapothu, S, Vinh B, et al. Attitudes towards chiropractic: a repeated cross-sectional survey of Canadian family physicians. BMC Fam Pract 2021;22:188.
3. Weis CA, Stuber K, Barrett J, et al. Attitudes toward chiropractic: A survey of Canadian Obstetricians. Journal of Evidence-Based Complementary & Alternative Medicine 2016;21(2):92-104.
4. Busse, JW, Jim, J, Jacobs, C, et al. Attitudes toward chiropractic: an analysis of written comments from a survey of north American orthopaedic surgeons. Chiropractic & Manual Therapies 2011, 19:25.
5. Beliveau PJH, Wong JJ, Sutton DA, et al. The chiropractic profession: a scoping review of utilization rates, reasons for seeking care, patient profiles, and care provided. Chiropractic & Manual Therapies 2017;25:35.
6. Triano JJ, Goertz C, Weeks J, et al. Chiropractic in North America: Toward a strategic plan for professional renewal – outcomes from the 2006 chiropractic strategic planning conference. J Manipulative Physiol Ther 2010;33:395-405.
7. Mior S, Gamble B, Barnsley J, Côté P, Côté E. Changes in primary care physician's management of low back pain in a model of interprofessional collaborative care: an uncontrolled before-after study. Chiropr Man Therap. 2013 Feb 1;21(1):6.
8. S Mior, Jan Barnsley, Heather Boon, Fredrick D. Ashbury & Robert Haig (2010) Designing a framework for the delivery of collaborative musculoskeletal care involving chiropractors and physicians in community-based primary care, Journal of Interprofessional Care, 24:6, 678-89,
9. Lisi AJ, Salsbury SA, Twist EJ, Goertz CM. Chiropractic Integration into Private Sector Medical Facilities: A Multisite Qualitative Case Study. J Altern Complement Med. 2018 Aug;24(8):792-800. doi: 10.1089/acm.2018.0218.
10. Busse JW, Riva JJ, Nash JV, Hsu S, Fisher CG, Wai EK, Brunarski D, Drew B, Quon JA, Walter SD, Bishop PB, Rampersaud R. Surgeon attitudes toward nonphysician screening of low back or low back-related leg pain patients referred for surgical assessment: a survey of Canadian spine surgeons. Spine (Phila Pa 1976). 2013 Apr 1;38(7):E402-8.
11. Boon HS, Mior S, Barnsley J, et al. The difference between integration and collaboration in patient care: Results from key informant interviews working in multiprofessional health care teams. J Manipulative Physiol Ther 2009;32:715-22.
12. Dykstra HP, Pollock N, Chakraverty R, et al. Managing the health of the elite athlete: a new integrated performance health management and coaching model. Br J Sports Med 2014;48:523-31.
13. Theberge N. The integration of chiropractors into healthcare teams: a case study from sport medicine. Sociology of health & illness [Internet]. 2008 Jan;30(1):19–34.

**Table – 1** **Demographic Characteristics of Respondents**

| **No. of respondents** 70 |
| --- |
| **Gender, n (%)**  Male 34 (48.57%)  Female 35 (50.00%)  Other 1 (1.43%) |
| **Years in practice, n (%)**  <5 yr 23 (32.86%)  5-10 yr 12 (17.14%)  11-20 yr 8 (11.43%)  >20 yr 27 (38.57%) |
| **Practice environment, n (%)**  Community 44 (62.86%)  Hospital-based 17 (24.29%)  Multidisciplinary 17 (24.29%)  Private practice 30 (42.86%)  Academic 16 (22.86%)  Other 3 (4.29%) |
| **Clinical area of interest, n (%)**  Family medicine 26 (37.14%)  Occupational medicine 5 (7.14%)  Emergency medicine 10 (14.29%)  Sports medicine 64 (91.43%)  Psychotherapy/Psychiatry 4 (5.71%)  Pediatrics 8 (11.43%)  Pain medicine 9 (12.86%)  Orthopaedics 15 (21.43%)  Physiatry 11 (15.71%)  Other 8 (11.43%) |
| **Experience working with a chiropractor, n (%)**  Yes 38 (54.29%)  No 22 (31.43%)  No, but open to working with a DC 10 (14.29%) |
| **Setting of work with a DC, n (%)**  Integrated Support Team (National Sport Organization) 16 (42.11%)  Professional Sports Team 11 (28.95%)  At a multisport games (i.e. Olympics, Pan Am Games) 20 (52.63%)  At a single sport competition (i.e. National Championship) 15 (39.47%)  Community-based sports medicine clinic 25 (65.79%)  Other 3 (7.89%) |


**Table – 2** **Responses to the Chiropractic Attitude Questionnaire Items (n = 70)**

| **Item** | **Strongly Agree**  **n (%)** | **Agree**  **n (%)** | **Undecided**  **n (%)** | **Disagree**  **n (%)** | **Strongly Disagree**  **n (%)** |
| --- | --- | --- | --- | --- | --- |
| Chiropractors promote unnecessary treatment plans: |  | | | | |
|  | 10 (14.29%) | 22 (31.43%) | 24 (34.29%) | 12 (17.14%) | 2 (2.86%) |
| Chiropractors provide effective therapy for some musculoskeletal conditions: |  | | | | |
|  | 15 (21.43%) | 38 (53.29%) | 8 (11.43%) | 4 (5.71%) | 5 (7.71%) |
| Chiropractors make excessive use of radiographic imaging: |  | | | | |
|  | 10 (14.29%) | 18 (25.71%) | 27 (38.57%) | 14 (20%) | 1 (1.43%) |
| Chiropractors provide a patient centered approach: |  | | | | |
|  | 3 (4.29%) | 31 (44.29%) | 27 (38.57%) | 5 (7.14%) | 4 (5.71%) |
| When I see patients who have attended a chiropractor, I often have to spend time correcting erroneous information that they have received: |  | | | | |
|  | 12 (17.14%) | 15 (21.43%) | 16 (22.86%) | 26 (37.14%) | 1 (1.43%) |
| Chiropractic manipulation of the neck is generally a safe therapy for patients: |  | | | | |
|  | 1 (1.43%) | 19 (27.14%) | 16 (22.86%) | 21 (30%) | 13 (18.57%) |

| Chiropractors can provide effective therapy for some non-musculoskeletal conditions: |  | | | | |
| --- | --- | --- | --- | --- | --- |
|  | 0 (0%) | 2 (2.86%) | 14 (20%) | 27 (38.57%) | 27 (38.57%) |
| Sport and exercise medicine physicians may risk professional liability if they refer a patient to a chiropractor: |  | | | | |
|  | 4 (5.71%) | 4 (5.71%) | 23 (32.86%) | 31 (44.29%) | 8 (11.43%) |
| Chiropractors can reduce patient overload for family physicians for patients with musculoskeletal complaints: |  | | | | |
|  | 3 (4.29%) | 24 (34.29%) | 18 (25.71%) | 12 (17.14%) | 13 (18.57%) |
| Chiropractors provide patients with misinformation regarding vaccination: |  | | | | |
|  | 7 (10%) | 14 (20%) | 36 (51.43%) | 10 (14.29%) | 3 (4.29%) |
| Chiropractic provides effective therapy for post-surgical rehabilitation: |  | | | | |
|  | 0 (0%) | 16 (22.86%) | 34 (48.57%) | 12 (17.14%) | 8 (11.43%) |
| Chiropractors lack sufficient clinical training: |  | | | | |
|  | 7 (10%) | 8 (11.43%) | 29 (41.43%) | 20 (28.57%) | 6 (8.57%) |
| Chiropractic care is a useful supplement to conventional sports and exercise medicine: |  | | | | |
|  | 8 (11.43%) | 32 (45.71%) | 17 (24.29%) | 6 (8.57%) | 7 (10%) |
| Chiropractors engage in overly aggressive marketing: |  | | | | |
|  | 13 (18.57%) | 17 (24.29%) | 24 (34.29%) | 15 (21.43%) | 1 (1.43%) |

| Chiropractic includes ideas and methods from which conventional medicine could benefit: |  | | | | |
| --- | --- | --- | --- | --- | --- |
|  | 2 (2.86%) | 24 (34.29%) | 29 (41.43%) | 8 (11.43%) | 7 (10%) |
| The results of chiropractic manipulation are due to the placebo effect: |  | | | | |
|  | 3 (4.29%) | 7 (10%) | 29 (41.43%) | 30 (42.86%) | 1 (1.43%) |
| Chiropractors treat in accordance with evidence-based practice: |  | | | | |
|  | 0 (0%) | 8 (11.43%) | 40 (57.14%) | 13 (18.57%) | 9 (12.86%) |
| Chiropractic has no role in the routine care of my patients: |  | | | | |
|  | 7 (10%) | 11 (15.71%) | 16 (22.86%) | 29 (41.43%) | 7 (10%) |
| Chiropractic breeds dependency in patients on short-term symptomatic relief: |  | | | | |
|  | 9 (12.86%) | 25 (35.71%) | 19 (27.14%) | 17 (24.29%) | 0 (0%) |
|  |  |  |  |  |  |
|  | **Very Good** | **Good** | **Undecided** | **Poor** | **Very Poor** |
| Overall, my impression of chiropractic is: |  | | | | |
|  | 6 (8.57%) | 26 (37.14%) | 17 (24.29%) | 13 (18.57%) | 8 (11.43%) |

**Table -3** **CAQ scores compared between medical specialties**

| **Data** | **Sample** | **Mean** | **Std Dev** | **Max** | **Min** | **Range** | **Overall positive view** | **Overall neutral view** | **Overall negative view** |
| --- | --- | --- | --- | --- | --- | --- | --- | --- | --- |
| CSP | 70 | 39.03 | 15.32 | 68 | 0 | 68 | 45.71% | 24.29% | 30.00% |
| North American  Orthopedic Surgeons (Busse et al., 2009) | 487 | 34.70 | 11.90 | 68 | 4 | 62 | 29.40% | 26.10% | 44.50% |
| Canadian Family Physicians  (Busse et al., 2021) | 251 (2010)  162 (2019) | 40.45  41.70 | 12.18  13.86 | 73  72 | 0  8 | 73  64 | 47%  49% | 27%  27% | 26%  24% |
| Obstetricians  (Weis et al., 2015) | 91 | 41.20 | 11.70 | 69 | 0 | 69 | 30% | 37% | 33% |

**Table – 4 CSPs Sources of Information on Chiropractic and Referral Practices**

| **Received chiropractic treatment as a patient, n (%)**  Yes 39 (55.71%)  No 31 (44.29%) |
| --- |
| **Self-rated knowledge of chiropractic, n (%)**  No knowledge 1 (1.43%)  A little knowledgeable 21 (30%)  Moderately knowledgeable 34 (48.57%)  Very knowledgeable 14 (20%) |
| **Did your medical training, prior to your sport and exercise medicine residency, expose you to information about chiropractic? n (%)**  Yes, overall favourable 3 (4.29%)  Yes, overall neutral 14 (20%)  Yes, overall unfavourable 21 (30%)  No 32 (45.71%) |
| **Did your sport and exercise medicine residency expose you to information about chiropractic? n (%)**  Yes, overall favourable 8 (11.43%)  Yes, overall neutral 25 (35.71%)  Yes, overall unfavourable 7 (10%)  No 30 (42.86%) |
| **How has your opinion of chiropractic been formed? n (%)**  Personal experience as a patient 31 (44.29%)  Patient feedback 51 (72.86%)  Family and friends 21 (30%)  Professors/supervisors/mentors 30 (42.86%)  Research literature 21 (30%)  Relationship with a specific chiropractor 37 (52.86%)  Media 2 (2.86%)  Residency 22 (31.43%)  Medical school 14 (20%)  Work experience (using chiropractic as a treatment for patients) 43 (61.43%)  I have no opinion on chiropractic 1 (1.43%)  Other 4 (5.71%) |
| **When were your opinions of chiropractic predominantly formed? n (%)**  Before medical school 12 (17.14%)  During medical school 11 (15.71%)  After medical school 45 (64.29%)  I have no opinion on chiropractic 2 (2.86%) |
| **How many patients do you refer for chiropractic care in a typical year? n (%)**  None 22 (31.43%)  1-10 22 (31.43%)  11-25 12 (17.14%)  26-50 7 (10%)  More than 50 7 (10%) |
| **If you do refer patients for chiropractic care, what drives the referrals? n (%)**  Patient request 38 (54.29%)  Non-response to medical treatment 18 (25.71%)  Literature supports chiropractic care for certain conditions 18 (25.71%)  Relationship with a specific chiropractor 26 (37.14%)  My own positive experience as a chiropractic patient 5 (7.14%)  I do not refer patients for chiropractic care 20 (28.57%)  Other 5 (7.14%) |

**Table – 5 Variables associated with Canadian sport and exercise medicine physicians’ attitudes towards chiropractic (n = 64)**

| **Variable** | **Unstandardized regression**  **coefficient from univariable**  **analysis**  **(95% CI)** | **p-value** | **Unstandardized regression**  **coefficient from multivariable**  **analysis**  **(95% CI)** | **p-value** |
| --- | --- | --- | --- | --- |
| Gender | 0.26 (-7.21 to 7.73) | 0.95 | 2.16 (-2.99 to 7.31) | 0.41 |
| Received chiropractic care | -0.57 (-7.98 to 6.84) | 0.88 | 3.03 (-2.48 to 8.53) | 0.28 |
| Number of patients referred for chiropractic care in a typical year | 6.34 (3.93 to 8.75) | 0.00 | 4.97 (2.83 to 7.11) | 0.00 |
| Information source for chiropractic ^a^ |  | | | |
| - Professors/supervisors/mentors | -1.74 (-9.17 to 5.68) | 0.64 | 5.13 (-0.71 to 10.96) | 0.08 |
| - Research Literature | -15.01 (-22.17 to -7.84) | 0.00 | -9.06 (-14.89 to -3.23) | 0.00 |
| - Media | -21.65 (-43.11 to -0.19) | 0.05 | -10.61 (-26.78 to 5.56) | 0.19 |
| - Residency | -6.01 (-13.80 to 1.79) | 0.13 | -4.29 (-10.50 to 1.92) | 0.17 |
| - Work experience (using chiropractic patient treatment) | 6.68 (-0.71 to 14.06) | 0.08 | -4.72 (-10.81 to 1.38) | 0.13 |
| Sport and exercise medicine residency included favourable information about chiropractic | 14.93 (3.94 to 25.92) | 0.01 | 9.07 (0.86 to 17.29) | 0.03 |
| Belief that adverse events are common with chiropractic care | -7.63 (-10.37 to -4.89) | 0.00 | -5.79 (-8.20 to -3.37) | 0.00 |

95% CI = 95% confidence interval

a = each sub-category was entered individually into the regression model as respondents could endorse multiple categories
